# Targeting disparate spaces: new technology and old tools

**DOI:** 10.3389/fpubh.2024.1366179

**Published:** 2024-04-23

**Authors:** Morgan M. Richey, John Bang, Vijay Sivaraman

**Affiliations:** ^1^Department of Epidemiology, University of North Carolina at Chapel Hill, Chapel Hill, NC, United States; ^2^North Carolina Central University, Durham, NC, United States

**Keywords:** equity, pollution, particulate matter, particulate matter <2.5 μm (PM) 2.5, African-American, policy, citizen science (CS), internet of things—IoT

## Abstract

A growing number of inexpensive, publicly available, validated air quality monitors are currently generating granular and longitudinal data on air quality. The expansion of interconnected networks of these monitors providing open access to longitudinal data represents a valuable data source for health researchers, citizen scientists, and community members; however, the distribution of these data collection systems will determine the groups that will benefit from them. Expansion of these and other exposure measurement networks represents a unique opportunity to address persistent inequities across racial, ethnic, and class lines, if the distribution of these devices is equitable. We present a lean template for local implementation, centered on groups known to experience excess burden of pulmonary disease, leveraging five resources, (a) publicly available, inexpensive air quality monitors connected via Wi-Fi to a centralized system, (b) discharge data from a state hospital repository (c) the U.S. Census, (d) monitoring locations generously donated by community organizations and (e) NIH grant funds. We describe our novel approach to targeting air-quality mediated pulmonary health disparities, review logistical and analytic challenges encountered, and present preliminary data that aligns with a growing body of research: in a high-burden zip code in Durham North Carolina, the census tract with the highest proportions of African Americans experienced worse air quality than a majority European-American census tract in the same zip code. These results, while not appropriate for use in causal inference, demonstrate the potential of equitably distributed, interconnected air quality sensors.

## Introduction

Inhalation of particulate matter (PM) from the ambient air is strongly associated with disease (1–6), mortality (7, 8), toxicity is known to be concentration dependent (9), and is highest among the smaller particle sizes such as PM_2.5_ (10, 11). Recent epidemiological studies have presented evidence that the burden of air pollution is unequally distributed, with differences along racial, ethnic, and class lines (12–14). In this commentary, we consider asthma, a persistent health inequity in the United States, the prevalence of which among African Americans was estimated to be 11.2%, compared to 7.6% among European Americans in 2019 (15).

Widespread measurement of particulate matter has expanded rapidly as inexpensive, internet-connected sensors are becoming widely available. But how will this new capacity be distributed? Historical health inequities in the United States and worldwide are often geographic, as illustrated by the phrase “the wrong side of the [railroad] tracks,” where industrial waste, air pollution and other health threats are concentrated in a less affluent (or segregated) area of a city, town, or other geographic region. We submit that the expansion of this new surveillance capacity is unlikely to include the large populations at the highest risk of the deleterious effects of poor air quality (16), perhaps due to the costs and requirement of the equipment, including stable power, Wi-Fi, and secure placement. Equitable distribution of air quality monitoring would involve longitudinal, fine-grained measurement of airborne pollutants in communities with elevated rates of diseases associated with these pollutants. We consider this as a unique opportunity for the scientific and academic community, as well as funders, to forge partnerships with community groups, citizen scientists, and state agencies to acquire and equitably distribute air quality monitoring devices, developing relationships that may have highly beneficial emergent properties (e.g., local knowledge of sources of emissions).

Ambient PM assessment is traditionally conducted by a field PM collection process with Federal Reference Methods (FRM) or EPA-approved FRM-equivalent approaches. These methods typically use a gravimetric filtration system run at a specific air flow rate for a specified run time or an indirect assessment of exposure level via particle counters using spectroscopic beams. While reliable and accurate, FRM studies for PM assessment are costly, requiring extensive preparation time and laboratory facilities to process specimens collected. Field measurements of any type can be challenging in historically high-burden areas due to a general distrust of researchers and lack of secure placement locations. The confluence of cost, access, and other logistical challenges has limited the collection of robust longitudinal air quality data in many communities. In North Carolina, high fidelity and longitudinal data is available from EPA and North Carolina DAQ stations. As there are a limited number of these stations, it is understood that substantial variation in air quality exists between stations. Researchers who wish to estimate pollutant burden in areas distant from air quality stations use interpolation techniques between stations (17, 18), remote sensing options including satellite imaging (19), or sample air quality at distinct points for a short period of time (20). These interpolations or short-duration monitoring efforts are often reasonable estimates of the air pollution burden for some areas but may under or overestimate it in others.

Health disparity research aims to provide actionable data to policymakers, who presumably use this data to develop harm reduction strategies. These data would ideally be collected in a wide area, be of high quality and granularity and would have regular and consistent measurement intervals. Data of this type could be used to measure concentrations of known carcinogens among groups experiencing health outcomes associated with exposure, evaluate policies intended to improve air quality, as well as serving as warning systems for acute spikes in emissions quality (e.g., wildfires). Emerging utilization of health disparity research includes action by citizen scientists to identify sources of pollutants, organizing and advocating for increased controls on emissions (21), and individuals using the information on air quality to adjust travel plans (22), and take steps to limit their exposure.

In this work, we present our template for collecting air quality information using new technology and reference methods, with a focus on communities at elevated risk of air-quality related pulmonary disease outcomes.

## Methods

### Outcome of interest: estimation of rate and geolocation of asthma exacerbation hot spots

Discharge data for all inpatient admissions and emergency room treat-and-release encounters in the State of North Carolina during the years 2012–2017 were obtained from the UNC SHEPS center. Using CDC guidance for identifying asthma encounters (23, 24), a case definition for probable asthma patients was constructed, using ICD9 and ICD10 diagnosis codes, as the period of interest spans the transition from ICD9 to ICD10. Any patient admitted to an inpatient unit or treated in an ER in North Carolina was eligible for inclusion in our sample. We then used the only geographic identifier available in this data: 5-digit billing zip codes, to restrict our sample to zip codes within the city of Durham. American Community Survey (ACS) estimates of the population of each zip code in each calendar year were aggregated over the study period, and five-year rates of asthma exacerbation in each zip code were calculated. The highest rate was in 27,701, downtown Durham, with a 5 year rate of 2.6 (95% CI: 2.4, 2.9) asthma exacerbation cases per 1,000 residents.

### Targeting geographic disparity: challenges and opportunities

**Figure 1:**
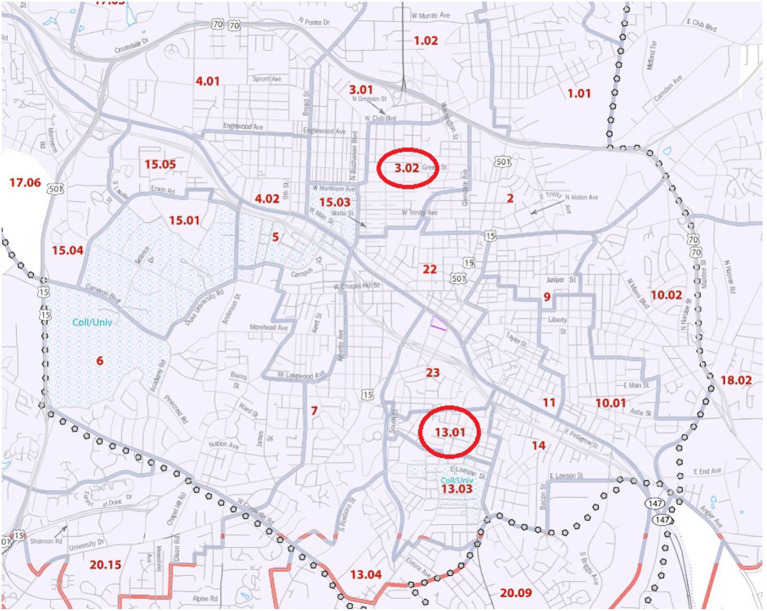
Location of Census Tracts 13.01 and 3.02 in Durham County, N.C. 2020 U.S. Census Tract Reference Map, Durham County, North Carolina.

Centering our monitoring approach on groups at known risk for PM-mediated pulmonary outcomes, we analyzed U.S. Census data and calculated the racial composition of census tracts with 50% or more of their area within zip code 27701. Among these tracts, there was a wide distribution of African American residents, ranging from 15–89%. We identified a census tract with the lowest proportion of African American residents (Census tract [CT] 3.02, 15%) and two with the highest proportion of African American residents (CT14, 89%, CT13.01, 89%) (Figure 1). We hypothesized that measures of PM_2.5_ would be highest in the primarily African American census tracts as measured by both the FRM personal environmental monitor (PEM) as well as the Wi-Fi-equipped purple air monitors (PA-II, Purple Air Inc., Draper UT). Within these census tracts, it was necessary to identify locations at which air quality monitors could be placed. We encountered both expected and unexpected challenges in gaining access to sites with both 120 V power, Wi-Fi access, and access for our field team.

#### Schools

We contacted schools in each census tract, as locating sensors on school grounds would allow for power, Wi-Fi, and secure access, but were unable to secure access. Informally, administrators shared their concern regarding the possibility of parental alarm if high PM_2.5_ levels were identified, resulting in curtailing outdoor time for children. This understandable but regrettable concern that identification of a problem would itself create a worse problem, is perhaps an under-appreciated challenge to community exposure measurement. It is important to note that these discussions occurred during the COVID-19 pandemic lockdowns, as educators, administrators, and parents were under extreme pressure; perhaps in a different setting this proposal would have been better received.

#### Parks

We contacted the park services in Durham city, as there were city parks in each census tract, but received no response to multiple inquiries. This non-response was especially unfortunate considering the central location of these parks, the availability of protected power, and ease of access.

#### Local businesses

We approached numerous retail businesses, and in most cases were unable to identify a responsible party who could approve placement of sensors. After navigating encounters with employees not empowered to make decisions, we left our contact information for the owner of the business. For the few owners who did respond, there was enthusiasm for the project, however either outdoor power was not available, or the locations in which it was available were not securable (e.g., ground-level outlets of a convenience store).

#### Community and faith-based organizations

Community and faith-based organizations ended up being our partners of choice; after being unable to identify a location in CT14 that would allow sensor placement, we identified a group in neighboring CT13.01, (with an almost identical proportion of African American residents) that allowed us to place sensors. Ultimately, the Vision of Africa community group in CT13.01, and the Beth El Synagogue in CT3.02 in July 2021 graciously allowed for placement of sensors on their property.

### Measurement strategy

We used two approaches to measuring the amount of PM_2.5_ in the two census tracts, the first using a Personal-Exposure Monitor, (PEM, SKC, Inc.) considered the reference standard, coupled with battery-operated air pumps (AirCheck XR5000, SKC, Inc.) at a flow rate of 2 L/min. PM_2.5_ samples by PEM units were collected on 37 mm Teflon filters that were pre-conditioned in a humidity controlled clean chamber for one day before and after field sampling to minimize the influence of humidity on the sample. Benefits of this approach include maximum sensitivity of measurements, but significant challenges are involved for long-term measurement due to the necessity of frequent filter/battery changes.

Our second approach involved the Wi-Fi-connected PM_2.5_ monitoring system (PA-II) that has been validated against FRM (25–27), including in urban settings in North Carolina (28). PA-II devices retail for between $220–$300, require 120 V power and can also store information on a SD card, or through the internet if connected through Wi-Fi. PA-II units were placed as far as 20 meters away from the WiFi routers used, with no appreciable loss of performance. Memory cards from units that were too far away from WiFi signals were collected daily, and replaced with a second card to avoid disruption in measurement.

### Physical location selection

#### Comparativeness of locations

Both locations are approximately 0.5 miles from Interstate Highway-40, a major source of vehicle originated PM_2.5_, and are 2.1 miles linear miles distance from each other. Census tract 3.02 has a perimeter of 3.5 miles and an area of 0.67 square miles. Census tract 13.01 has a perimeter of 2.9 miles and an area of 0.28 square miles. Both tracts are primarily residential, with tree covering, and both feature a school and park. Census tract 3.02 appears to have a larger proportion of area covered with trees due to a nature preserve located in the north-east corner.

#### Duration of measurement

Both PEM and PA-II sensors were placed on November 22, 2020, and collected data for 5 days before a PEM unit was lost from one of the sites. After replacing the PEM unit, sensors ran for another 5 days between November 26 and December 3, 2020. This 2 week measurement period is a common minimum monitoring period in air quality studies, but is not presented as a measure of year-long air quality.

## Results

During the surveillance period in November–December 2020, the levels of measured PM2.5 by the PA-II wireless sensor and PEM units identified higher PM2.5 burden in CT13.01 compared to CT3.02 (Table 1). The average weight of the PM_2.5_ collected by the PEM unit in CT 13.01 during the study period was forty-nine percentage points larger than that collected in CT3.02. Measurements from the PA-II unit during the same period also showed higher concentrations of PM2.5 in CT13.01, as well as more days spent in the “unhealthy” air quality index compared to CT3.02.

**Table 1:** Particulate matter measurements from PA-II and federal reference standard: November–December 2020, city of Durham, N.C.

Period 1					Period 2				
Purple Air PA-II					Purple Air PA-II				
Census Tract 13.01			Census tract 3.02		Census tract 13.01			Census tract 3.02	
Daily avg	PM_2.5_	AQI	PM_2.5_	AQI	Daily avg	PM_2.5_	AQI	PM_2.5_	AQI
11/22/2020	76.2	Unhealthy	70.5	Unhealthy	11/29/2020	28.4	Moderate	28.8	Moderate
11/23/2020	76.2	Unhealthy	13.5	Moderate	11/30/2020	28.4	Moderate	28.7	Moderate
11/24/2020	76.1	Unhealthy	41.0	Unhealthy: Sensitive Groups	12/1/2020	28.4	Moderate	28.7	Moderate
11/25/2020	76.0	Unhealthy	59.5	Unhealthy	12/2/2020	28.3	Moderate	28.6	Moderate
11/26/2020	75.9	Unhealthy	42.4	Unhealthy: Sensitive Groups	12/3/2020	28.3	Moderate	28.5	Moderate
PEM (both periods)									
Location	PM_2.5_ Concentration						PM_2.5_ WeigHT		
Census tract 13.01	462 μg/5.886 m^3^						0.462 mg		
Census tract 3.02	310 μg/5.886 m^3^						0.310 mg		

Note: Daily PM_2.5_ levels are reported as the average of PA-II readings between 12:00am and 11:59pm.

## Discussion

In this work, we demonstrate one approach to operationalizing the ideal of equity in the expansion of automated, local air quality measurements by centering data collection on populations that bear a disproportionate pulmonary disease burden. We apply a pragmatic and economically lean approach using a small NIH grant, low-cost sensors, state-collected hospital discharge data, publicly available U.S. Census demographic information, collaboration with local businesses and community organizations, and a simple analytical approach. Our results are aligned with previous research that have found that racially minoritized populations experience greater concentrations of PM_2.5_ and poorer AQI in their neighborhoods compared to non-minoritized populations (12–14, 18, 29, 30).

Our approach was novel, in that we identified hot spots of asthma exacerbation by 5-digit zip code from billing data, then identified disparate spaces by race in the census tracts within. The contrasting of a census tract with a high proportion of African American residents with one with the highest proportion of European American residents aligns with the concept of centering data collection on populations known to be at risk of a given outcome; in this case asthma. We encountered significant challenges, primarily revolving around placing sensors in a location that had access, power, and internet services. Duration of monitoring was also curtailed after the loss of a PEM monitor, highlighting the challenges that monitors that require regular servicing (battery, filter changes) involve. Future endeavors could consider which location types, including residential, business or community groups, allow for the longest and highest fidelity of measurement. In many cases, investigators could consider whether small payments, or other forms of compensation, may be effective in retaining access to monitoring sites. We found that building a supportive partnership with community groups was critical to the success of this work.

It is important to note that this work is not designed to show a causal association between PM_2.5_ concentration and asthma exacerbation. Instead, we demonstrate a template approach to data collection that avoids the trap of representativeness (31), instead pursuing a strategy of pragmatic utilization of free or low-cost resources to focus on a population that is at high risk of poor cardiopulmonary outcomes. We propose that a sensor network of this type, carefully positioned, could generate actionable data to support causal inference in pulmonary outcome inequity research. With the help of grant funders, community leaders, academic institutions and faith-based organizations, the future of air quality surveillance can be one that includes those currently experiencing its effects.

## Data availability statement

The hospital and emergency-room discharge data used in this work is available from the Cecil B. Sheps Center for Health Service Research. The raw data supporting the conclusions of this article will be made available by the authors, without undue reservation.

## Ethics statement

This study was reviewed by the Institutional Review Board of the University of North Carolina at Chapel Hill and determined not to be human-subjects research (IRB #19-2185).

## Author contributions

MR: Writing – original draft, Visualization, Methodology, Investigation, Formal analysis, Data curation, Conceptualization. JB: Writing – review & editing, Writing – original draft, Supervision, Project administration, Methodology, Formal analysis, Conceptualization. VS: Writing – review & editing, Supervision, Project administration, Methodology, Funding acquisition, Conceptualization.
